# Supplementary material for: Difference in gaze control ability between low and high skill players of a real-time strategy game in esports
Source: PLoS One. 2022 Mar 18;17(3):e0265526. doi: 10.1371/journal.pone.0265526 (PMC8933040; doi:10.1371/journal.pone.0265526)
Supplement: S3 File — (DOCX) [file pone.0265526.s003.docx]

***PLoS One* Supporting Information file S3**Article title: Difference gaze control ability between low and high skill players of a real-time strategy game in esports
Authors: Inhyeok, Jeong., Kento, Nakagawa., Rieko, Osu,. Kazuyuki, Kanosue.


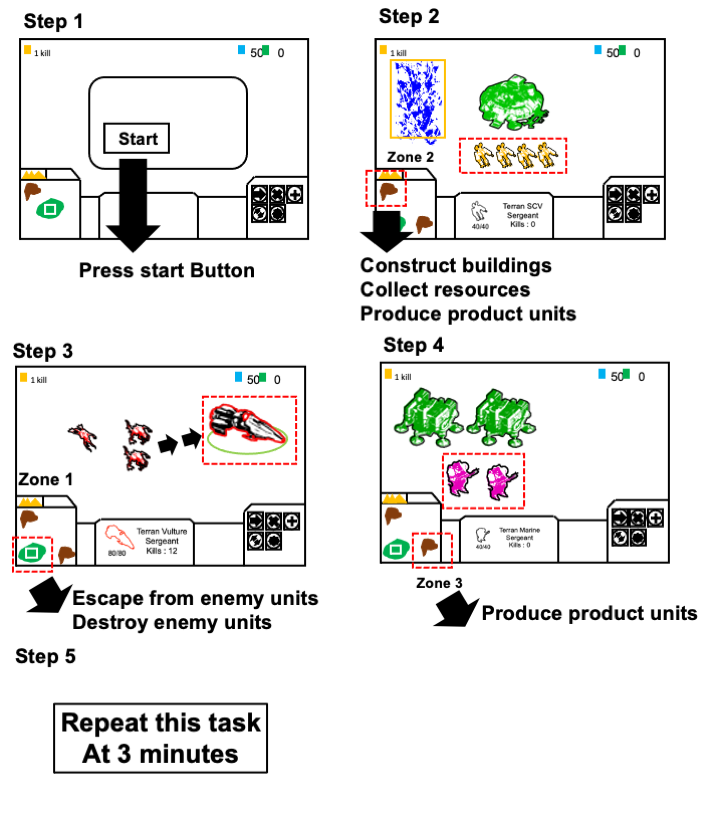


**Figure C. Detail of Hard Task**
