# Supplementary material for: Difference in gaze control ability between low and high skill players of a real-time strategy game in esports
Source: PLoS One. 2022 Mar 18;17(3):e0265526. doi: 10.1371/journal.pone.0265526 (PMC8933040; doi:10.1371/journal.pone.0265526)
Supplement: S4 File — (DOCX) [file pone.0265526.s004.docx]

***PLoS One* Supporting Information file S4**Article title: Difference in gaze control ability between low and high skill players of a real-time strategy game in esports
Authors: Inhyeok, Jeong., Kento, Nakagawa., Rieko, Osu,. Kazuyuki, Kanosue.


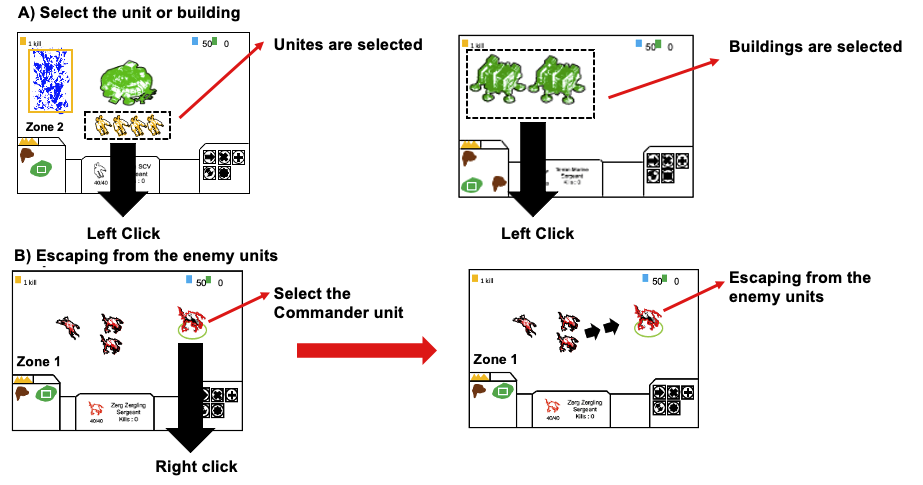


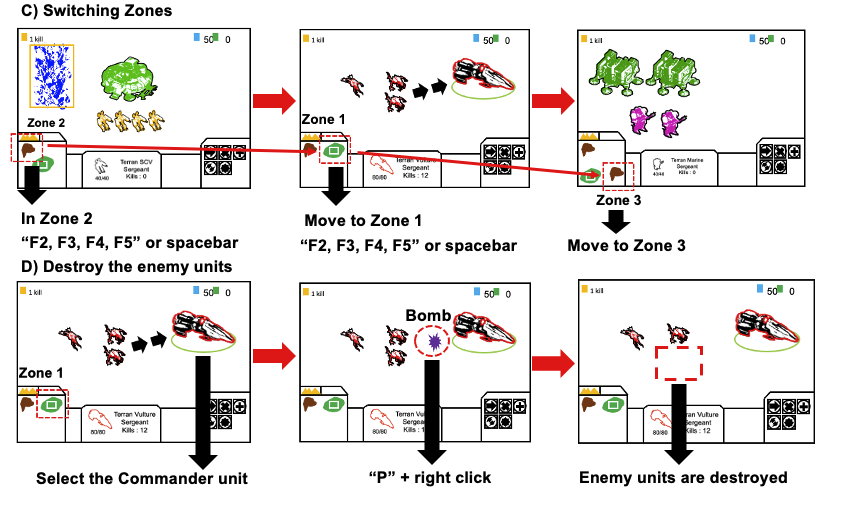


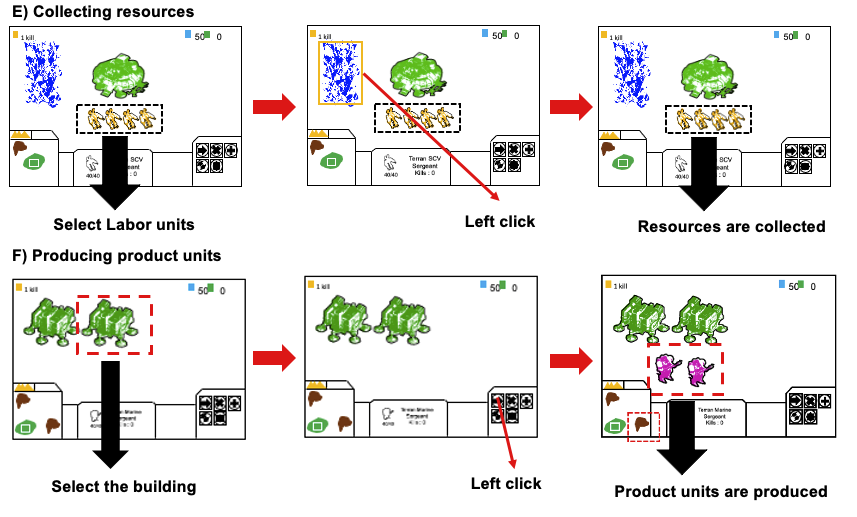


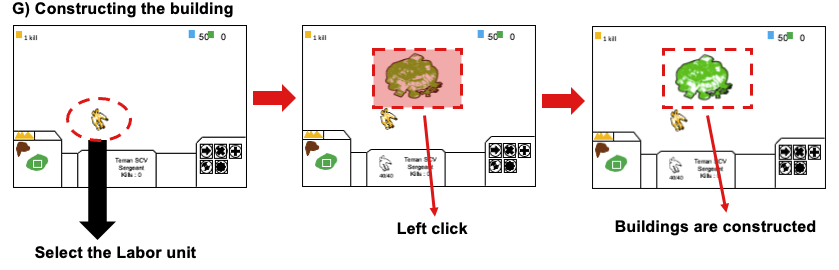


**Figure D. Detail of operation method.** Each parts of figure shows the specific steps of operation method.
