# Supplementary material for: Difference in gaze control ability between low and high skill players of a real-time strategy game in esports
Source: PLoS One. 2022 Mar 18;17(3):e0265526. doi: 10.1371/journal.pone.0265526 (PMC8933040; doi:10.1371/journal.pone.0265526)
Supplement: S5 File — (DOCX) [file pone.0265526.s005.docx]

***PLoS One* Supporting Information file S5**Article title: Difference in gaze control ability between low and high skill players of a real-time strategy game in esports
Authors: Inhyeok, Jeong., Kento, Nakagawa., Rieko, Osu,. Kazuyuki, Kanosue.


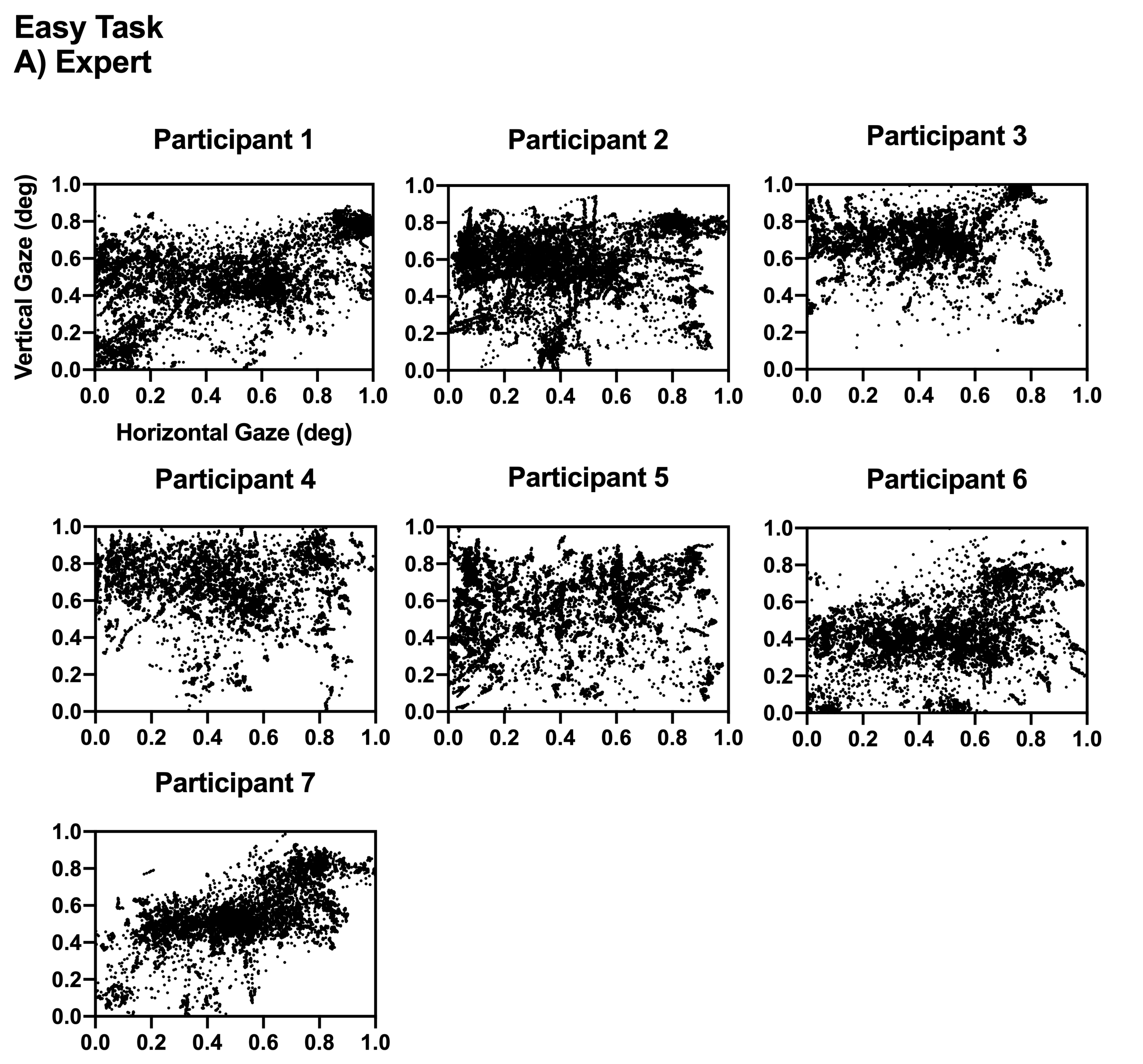


**Fig A. Raw data of Easy Task, Expert.**

**
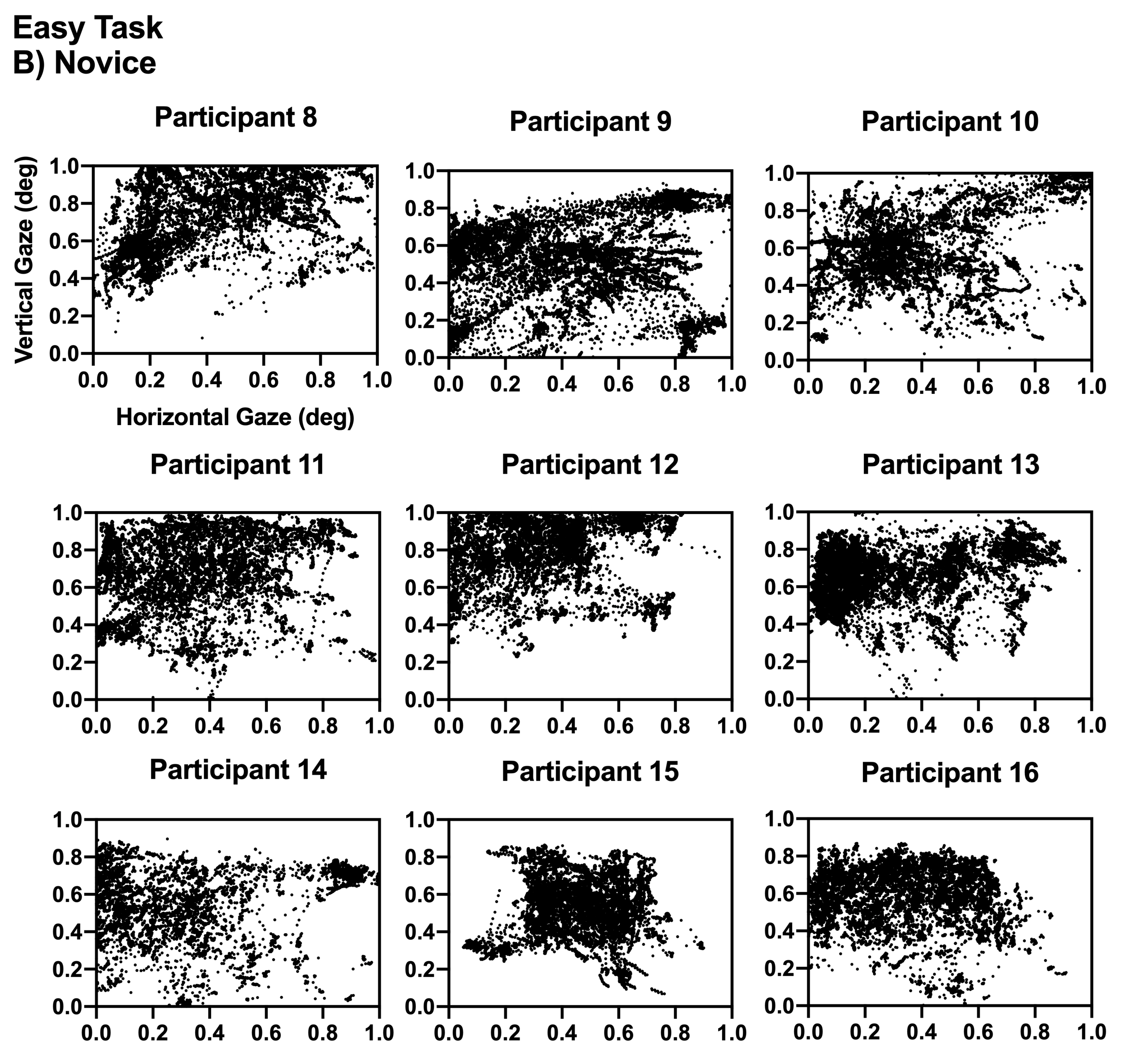
**

**Fig B. Raw data of Easy Task, Novice.**

**
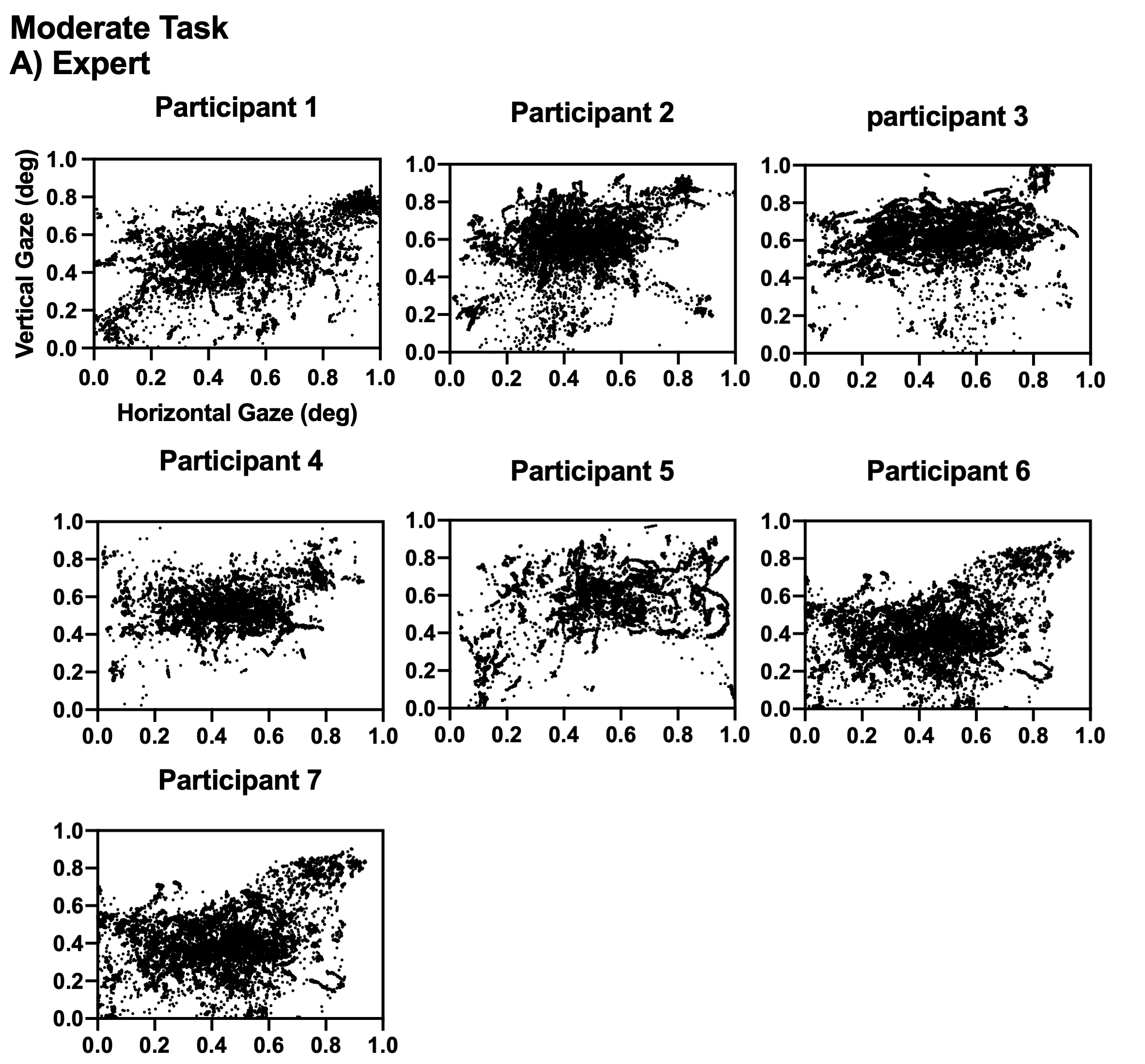
**

**Fig C. Raw data of Moderate Task, Expert.**

**
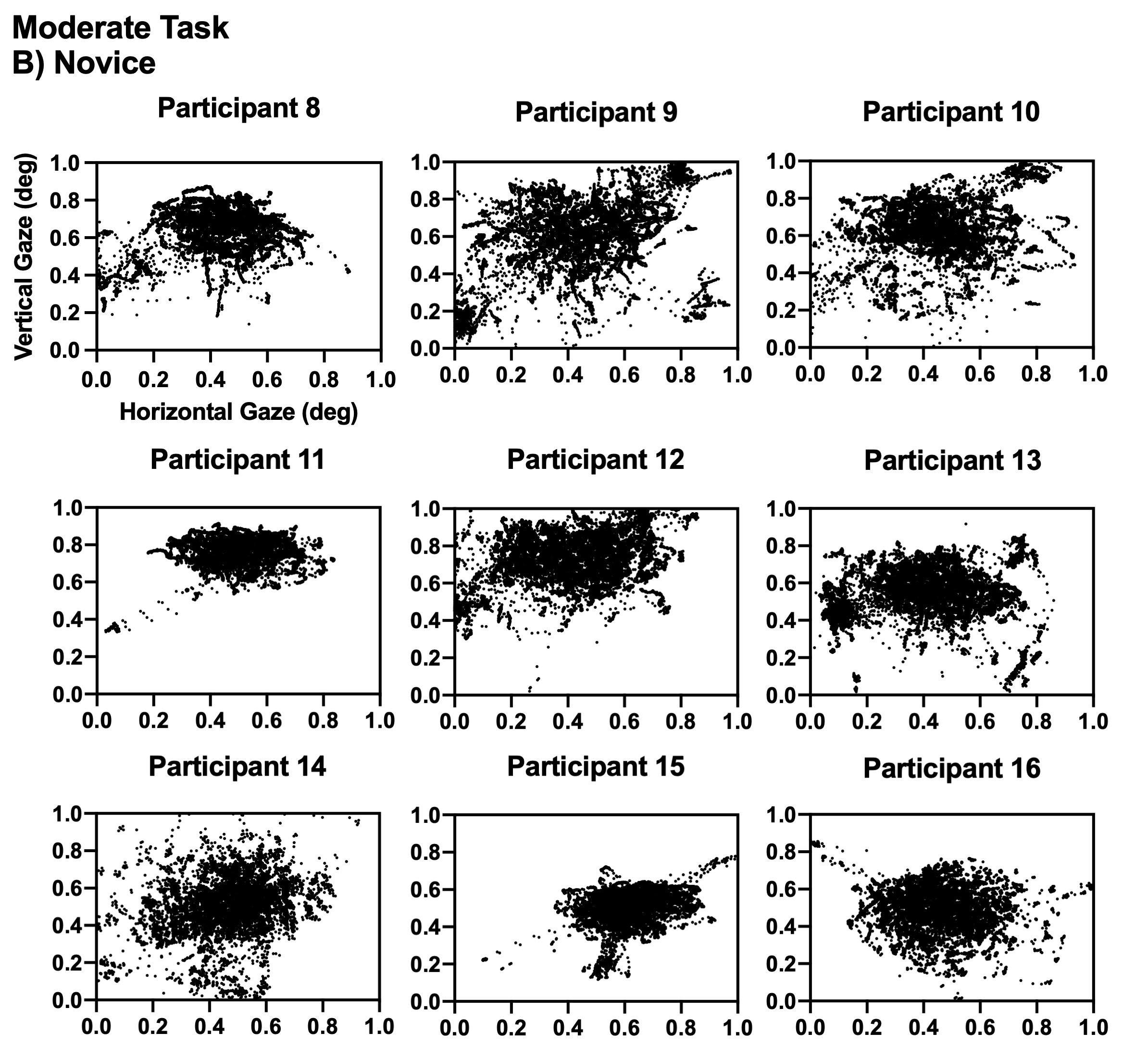
**

**Fig D. Raw data of Moderate Task, Novice.**

**
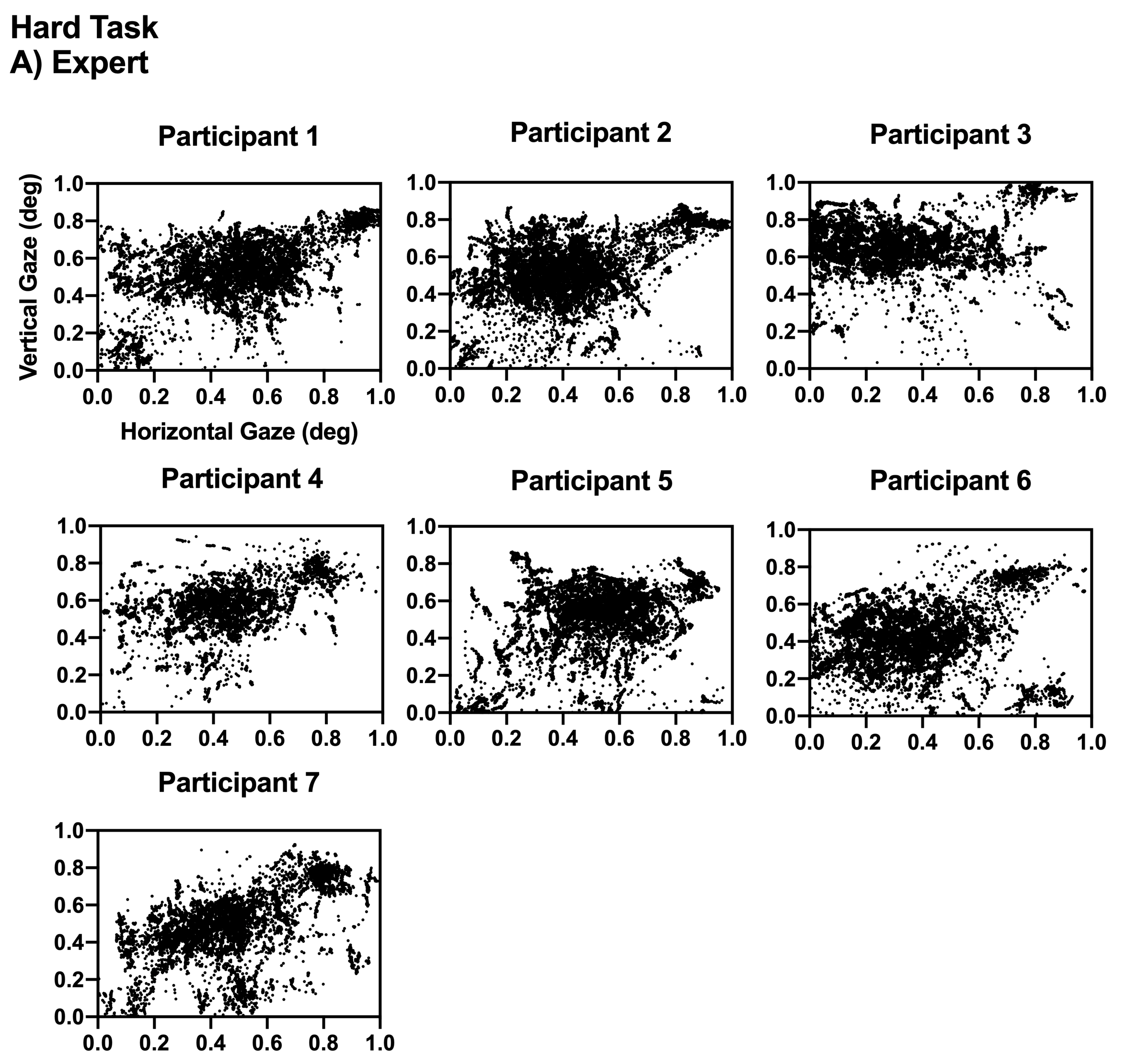
**

**Fig E. Raw data of Hard Task, Expert.**

**
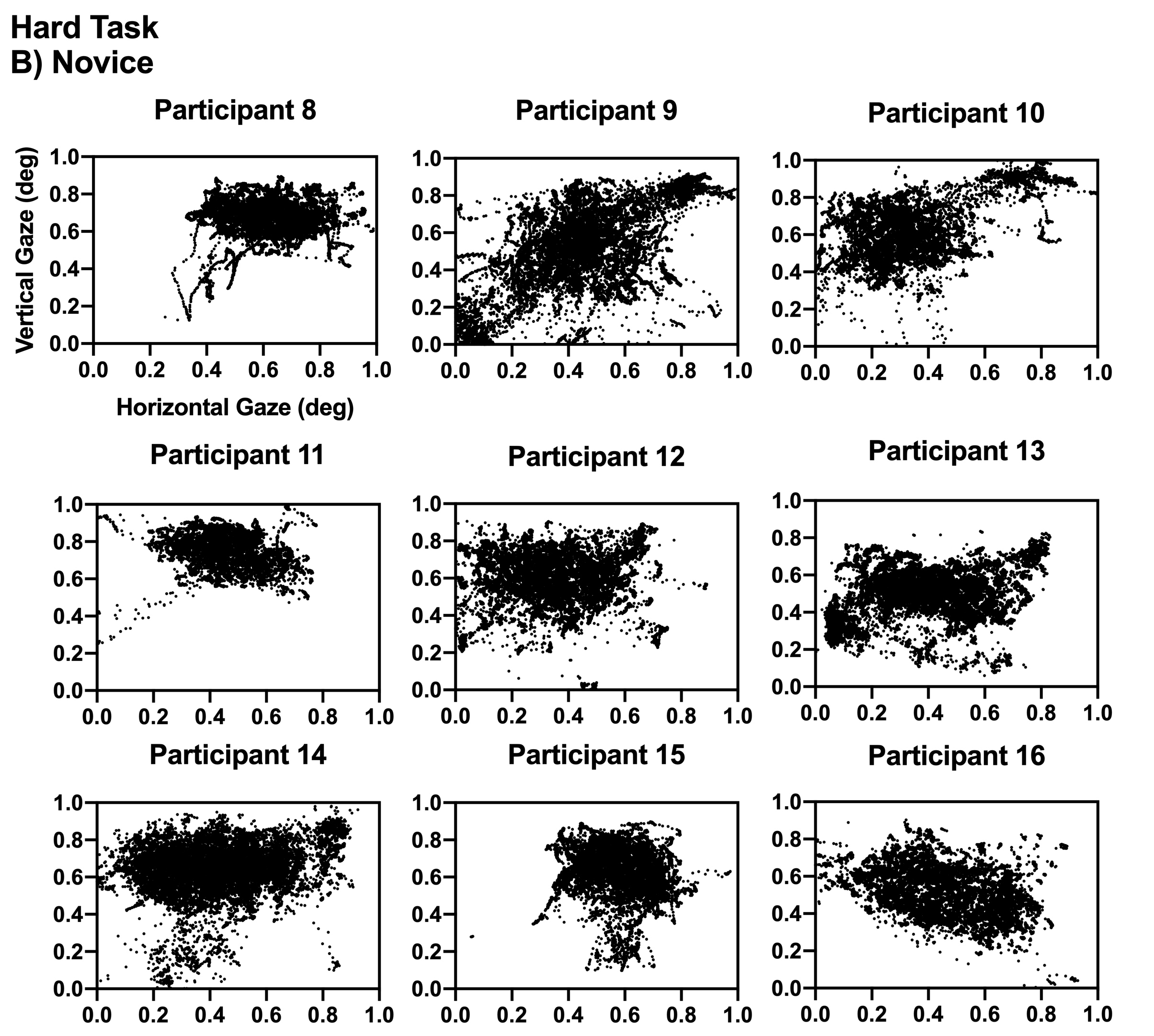
**

**Fig F. Raw data of Hard Task, Novice.**
