# Supplementary material for: Difference in gaze control ability between low and high skill players of a real-time strategy game in esports
Source: PLoS One. 2022 Mar 18;17(3):e0265526. doi: 10.1371/journal.pone.0265526 (PMC8933040; doi:10.1371/journal.pone.0265526)
Supplement: S6 File — (DOCX) [file pone.0265526.s006.docx]

***PLoS One* Supporting Information file S6**Article title: Difference in gaze control ability between low and high skill players of a real-time strategy game in esports
Authors: Inhyeok, Jeong., Kento, Nakagawa., Rieko, Osu., Kazuyuki, Kanosue.

**Raw data of each participant:** Expert (224, 212, 153, 225), Low Skill (146, 176, 113,141)

**Power calculation for unpaired t-test between Expert (n = 4) vs. Low Skill (n = 4)**

**t test:** Means: Difference between two independent means (two groups)

**Analysis:** Compute required sample size

**Input:** Tail(s) = Two

Effect size d = 1.91

α err prob = 0.05

Power (1-β err prob)) = 0.9

Allocation ration N2/N1 = 1

**Output:** Noncentrality parameter δ = 3.57

Critical t = 2.17

Df = 12

Sample size group 1 = 7

Sample size group 2 = 7

Total sample size = 14

Actual power = 0.905
