# Supplementary material for: Difference in gaze control ability between low and high skill players of a real-time strategy game in esports
Source: PLoS One. 2022 Mar 18;17(3):e0265526. doi: 10.1371/journal.pone.0265526 (PMC8933040; doi:10.1371/journal.pone.0265526)
Supplement: Attachment — Submitted filename: response letter.docx [file pone.0265526.s007.docx]

**Response to Editor and Reviewers**

Dear Academic Editor and Reviewers,

Thank you very much for your feedback, comments and suggestions. Our responses to your comments are as follows:

**Editor:**

Comment 1: Please ensure that your manuscript meets PLOS ONE's style requirements, including those for file naming.

**Response:** The style of the manuscript has been corrected. To be specific, the name of the figures file have been changed to follow PLOS ONE requirements.

Comment 2: Please provide additional details regarding participant consent.

**Response:** We have corrected as follows:

**Change:** [P5L88-90] Before the experiment, we verbally provided information about the contents and concepts of this research along with the instruction documents. After that, we obtained verbal informed consent from all subjects.

Comment 3: We note you have included a table to which you do not refer in the text of your manuscript. Please ensure that you refer to Table 2 in your text.

**Response:** We have referred to Table 2 as follows:

**Change:** [P10L164] The above mentioned conditions for game over are summarized in Table 2.

Comment 4, 4.1, 4.2: We note that Figures 1, 2. S1, S2, S3 in your submission contain copyrighted images.

**Response:** Thank you for your consideration of copyrighted images. Figure 1, 2 and S1, 2, 3, 4 has been replaced to illustration.

**Reviewer #1:**

**Recommendations:** The authors present a study that examines the behavioral and gaze differences between novice and expert StarCraft players. The research question is of interest to the field, the methodology and statistical analyses are generally sound and the findings are interesting. However, there are major revisions that need to be addressed in the introduction and justification of the study, the reporting of results and the discussion of the findings that would need to be addressed before the manuscript could be considered for publication.

**Response:** Thank you for your recommendations. The introduction and the reporting of results and　discussion have been reworked.

Also, the grammar is very poor despite the authors report that it had been examined for proper use of the English language. In many instances, wording is awkward and the plural of a word is required and not used among other grammatical issues. This significantly takes away from the readability of the paper and presentation of the work and a thorough edit of the manuscript’s grammer by someone whose first language is English is required.

**Response:** Thank you for your suggestion. According to your suggestion, the manuscript has been　proofread by a professional proofreading service again.

**INTRODUCTIONS:**

P2L35: Please change ‘e-sports’ to ‘esports’ throughout manuscript, unless starting a sentence, which in that case, ‘Esports’ is correct.

**Response:** We are sorry for using incorrect words. The word “e-sports” has been replaced with “esports”　and “Esports”

P3L39: esports is not a subcategory of AVG, this would imply that all esports are AVGs. Instead, AVGs, when played competitively and/or professionally, can be considered esports. Please reword.

**Response:** Thank you for your suggestion. Definition of esports has been reworded and a reference has been added as follows:

**Change:** [P2L35-36] Irrespective of game genre, when a game is played competitively and professionally, it can be considered esports [2].

P3L40-41: It isn’t the expanding market that has led to research on cognition in esports. I would reword this to highlight that esports players are often considered cognitive athletes and it’s this foundation , in conjunction with the increased popularity of esports that has led to an increase in research attention toward the cognitive benefits associated with esports and VG more broadly. See and reference Campbell et al., 2018.

**Response:** We appreciate the suggestion. The reason why research on esports has been expanded has been added.

**Change:** [P3L39-40] In addition, the increasing popularity of esports has resulted in further research on the cognitive benefits associated with esports and video games [3]

General Intro : Missing a key reference regarding superior info processing and task-switching abilities of AVG’s compared to Non Gamers that would support the authors introductory arguments.

**Response:** Thank you for your suggestion. According to your suggestion, a key reference has been added

as follows:

**Change:** [P3L40-41] Kowal et al. [16] found that the task-switching ability of action video game (AVG) players is higher than that of non-AVG players.

P3L49: AVG should be AVGs (plural). Also to this line…what is the difference between cognitive and visual function? Visual function as in a change in the function of the retinal cells? The visual cortex? Or do you mean visual perception here? Would be careful with wording here as if it’s a change in saccade pattern for example, this isn’t a visual function change but an adaptation to a change in cognitive function or strategy.

**Response:** Thank you for your suggestion. AVG has been revised to AVGs. We are sorry for using　ambiguous words (cognitive and visual function). We removed the ambiguous “visual function”　in the whole paragraph. Additionally, the definition of “cognitive” has been added.

In the current study, cognitive function refers to the mental process involved in obtaining information and knowledge.

**Change:** [P3L43-44] In addition, a recent meta-analysis also found that video game practice has positive effects on cognitive function [14] (the mental processing required to obtain information and knowledge [6]).

P4L60-66: Why is looking at in-game saccade behaviour important? What evidence is there from other cognitive aspects that results in clinical testing and in game testing might result in different conclusions being made? This is a key point the authors must address in their justification of this study.

**Response:** Thank you for your suggestion. In esports, especially in real-time strategy (RTS) games, multiple stimuli appear simultaneously and players are required to judge how to respond to those stimuli. For high RTS performance, players need to check multiple information sources, switching from one to the other as fast as possible. In this situation, players should make fast saccadic gaze movements. In order to test this hypothesis, ordinary clinical testing using a simple task would not be appropriate because simple tasks present less stimuli and do not require the degree of multitasking required in RTS. Indeed, Murphy and Spencer (2009) argue that there was no significant difference between esports players and non-esports players in visual attention skill, which was verified only in a simple attention blink task used in the laboratory

. The possible reason why this previous research did not find better visual ability in esports players would be that the simple laboratory task may be too easy because multitasking was not incorporated in the task. For this reason, in the current study we set the task to be like a real game in order to detect the strategy esports players use to control their gaze during game playing.

Information about the importance of measuring saccade behavior and the difference between a clinical test and a gaming test based on the evidence has been added as follows:

**Change:** [P3L51-P4L67] Indeed, in RTS, multiple information streams appear simultaneously in different locations on the monitor screen, and players must absorb and select information of importance to them. Based on the information obtained, they must judge how to make an appropriately timed response to the stimuli. Thus, multitasking ability could be the key element underlying higher performance in RTS. RTS players must move their gaze over multiple areas on the monitor to win the game. Therefore, esports players would use more ballistic gaze movements, called saccade, to enable them to process multiple stimuli more quickly. However, the characteristics of gaze strategy in esports players are still unknown. Therefore, the purpose of the current study was to examine the gaze control of RTS expert players. We hypothesized that RTS experts in esports would show superior gaze control strategy (wide and fast saccadic gaze movement) which is evaluated by measuring gaze distribution and saccadic movements. Clarifying the gaze control strategy used by successful esports players would be beneficial for esports coaching, and also for expanding esports markets.

Murphy and Spencer [17] argue that there was no significant difference between esports players and non-esports players in visual attention skill; this conclusion was verified by using only a simple attention blink task in a laboratory, which was unrealistic because there was no multitasking involved. Therefore, in the current study for analyzing how esports players control their gaze while playing a game, we set them a task which was similar to what occurs in real RTS games; we required players to exhibit higher multitasking abilities, and to cope with increasing task difficulty.

P4L67: Starcraft is a real time strategy game (RTS). As it is not considered an AVG, previous arguments around AVGs and enhanced cognition or saccade performance do not justify examining Starcraft here. The authors would need to rework their entire introduction to justify examining gaze patterns for this style of video game, as opposed to basing all of their evidence around AVGs.

**Response:** We appreciate your comments. We changed the genre of StarCraft from AVG to RTS. Additionally, information about cognitive functions in RTS players and the reason why it is important to focus on gaze patterns has been added as follows:

**Change:** [P3L45-50] The real-time strategy (RTS) game is another kind of video game. In RTS games players are required to multi-tasking using complex strategies. AVG and RTS have similar positive effects on cognitive processes (both AVG and RTS improve reaction time and problem-solving ability) [10]. Meanwhile, Dobrowolski et al. [4] found that RTS players have superior cognitive function compared to that of AVG players. Indeed, RTS game training improves task switching ability [18]. In order to switch between tasks, it is necessary to accurately perceive stimuli presented on the monitor screen through proper gaze control.

P4L70-71: Current hypothesis is not an actual hypothesis. Given the purpose is to examine gaze control strategy between novices and expert SC players, the hypothesis should explicitly state who will be expected to show superior gaze control strategy and what metric will indicate this. Please edit hypothesis.

**Response:** We agree with your suggestion. We have edited the hypothesis as follows:

**Change:** [P4L58-59] We hypothesized that RTS experts in esports would show superior gaze control strategy (wide and fast saccadic gaze movement) which is evaluated by measuring gaze distribution and saccadic movements.

**METHODS:**

N=16, N Experts = 7, N Novices = 9

The N's seem very low. Can the authors provide a power analysis justifying the minimum number of participants required to be able to meaningfully detect the effects they hope to detect?

**Response:** Thank you for this suggestion. Information about power analysis has been added.

**Change:** [P5L78-84] Before starting the experiment, we performed a power analysis to estimate the required sample size (RStudio version 1.3). The sample size was calculated using preliminary experimental data related to the distribution of gaze movements of esports experts (Expert) and players with lower skills (Low Skill) (effect size: 0.51; α level: 0.05; power (1-β error probability): 0.80). As a result, the calculated necessary number of participants was seven in each group. According to the result of this power analysis, sixteen participants (15 male, 1 female; 7 Expert, 9 Low Skill; mean age, 22.4 years; age range, 18-28 years) with experience playing StarCraft participated in the present study.

P5L82: Authors indicate that all participants have experience playing Starcraft. This being the case, it is incorrect to call any of the participants ‘Novices’ and instead, the groups should be separated based on skill level (Expert or high skill vs. Low Skill).

**Response:** Thank you for your consideration. All ‘Novice’ have been changed to ‘Low Skill’. Along with this change, “expert” was also changed to “Expert”.

Did the authors control for other video games that individuals played? Other genres? How often they currently spend playing video games (i.e., hours per week). These are all important considerations in defining the groups that should be considered and added where appropriate. Otherwise, these aspects should be included within the limitations section of the discussion.

**Response:** Unfortunately, we did not control for other video games in other genres and collect the participants’ history of esports play. Lack of information of other esports play history could affect the participants’ properties. Additionally, we could not rule out the possibility that other esports play history has affected the difference between the Expert and the Low Skill. A statement about playing history has been added to the limitation section.

**Change:** [P22L393-395] we did not record the participants’ experience in playing other esports or their years of esports experience. Therefore, we cannot rule out the possibility that a history of playing other esports affected the difference in gaze movement between the Expert and the Low Skill players.

P10L168-172: APM does not measure speed of hand movement per se. Hands would have to be kinematically tracked using 3d Motion capture hardware and software. Please revise wording here.

**Response:** We strongly agree with your opinion. Thus, “speed of hand” has been changed to “number of clicks”.

**Change:** [P11L178-179] In order to compare the number of clicks between the Expert and Low Skill players,

P11L178: I would like to see the distribution of monitor sizes used by participants in the study reported. It’s very easy to say there is no difference but what test was used? Were the monitor sizes across participants normally distributed. I would guess not. How did the different metrics pay out for different monitor sizes?

**Response:** Thank you for your question. Data about monitor size followed homogeneity but not normality. For this reason, Wilcoxon Rank-Sum test was used to check significant difference in monitor size between Expert and Low Skill. No significant difference in monitor sizes between the groups was found (p = .30). Information of monitor sizes (means and SD) has been added.

**Change:** [P11L185-187] There was no significant difference in the selected monitor size between Expert players (average of monitor size: 27.00 inch ± SD 3.87) and Low Skill players(average of monitor size : 28.77 inch ± SD 4.35) (Wilcoxon Rank-Sum test, *p* = .30).

P11L179-180: How was head position maintained at 40 inches? How was this ensured or tracked? What was the variability in head position across 3 min trials? These need to be explained as gaze performance conclusions can be confounded by this.

P11L179-180: What was the variability in head position across 3 min trials? These need to be explained as gaze performance conclusions can be confounded by this.

**Response:** We confirmed the initial distance between each participant and the monitor was 40 inches before starting the task. Since we instructed participants to keep the current position of their heads while sitting on the chair, the head position was unlikely to move very much. However, as we did not track the actual head position, we cannot strictly control the movement of the head. This information has been added to the limitation section.

**Change:** [P11L187-188] Before the task started, we measured the distance (40 inches) between each participant’s head and their monitor. After measuring the distance, we instructed participants to keep the position of their head steady.

**Change:** [P22L395-397] Furthermore, we were not able to strictly control participants’ head movements, and the possibility that the difference in gaze movement was caused by head movement cannot be excluded.

P13L222: Were data tested for normality and homogeneity of variance? Please provide these details.

**Response:** Thank you for your suggestion. As we did not check the normality nor homogeneity of variance in the previous version, we have analyzed them by Levene’s test and Shapior-Wilk’s test in this revision. We found that SD of gaze distribution (vertical gaze), AOI, and saccade characteristic (saccade velocity) did not follow normality and homogeneity of variance. Therefore, these data were reanalyzed by a non-parametric method (Kruskal Wallis test and Wilcoxon rank sum test with Bonferroni’s correction). We have added information about normality and homogeneity of variance as follows:

**Change:** [P14L233-239] Homogeneity of variance was tested with Levene’s test and normality of variance was tested with Shapior-Wilk’s test. As a result, SD of gaze distribution (vertical gaze), ratio of fixation in each area (AOI), and saccade velocity did not follow a normal distribution of variance and show homoscedasticity. For this reason, these data were calculated by a non-parametric method. When the Kruskal-Wallis test detected significance, the Wilcoxon Rank-Sum test was performed to find the difference between each group of Task difficulties (Bonferroni’s correction (*p* < .05 divided by 3 tests: significance threshold at .017)).

[P18L309-311] The saccade velocity of Expert players was significantly faster than that of Low Skill players (Wilcoxon Rank-Sum test, *p* = .02).

**RESULTS:**

P14L236: How are data presented: Means +- SD or SE or 95%CI??? Please state.

**Response:** We are sorry for omitting the statement. In the behavioral data section, data were presented as means ± SD.

P15L265: Incorrect to say ‘Simple main effect analysis found that the AOI of the Easy Task was significantly larger than that of the Hard Task (p = .02)’ Instead, Proportion of time gaze stayed in area 3 was greater for easy vs hard task. Please revise wording around this finding.

**Response:** Thank you for your suggestion. In this paragraph (Area of Interest), all data have been recalculated with non-parametric analysis. Thus we have reworked all statistics descriptions.

**Change:** [P16L280-P17L288] The proportion of time that the gaze stayed on each AOI (ratio of gaze distribution) is shown in Figure 5. In Area i, there was a significant difference of skill level (Wilcoxon Rank-Sum test, *p* < .001) while there was no significant difference between Task difficulty. In Area ii, there was no significant difference in skill level or Task difficulty. In Area iii, a significant difference of skill level was found (Wilcoxon Rank-Sum test, *p* = .02) while there was no significant difference in Task difficulty. In Area iv, there was no significant difference in skill level or Task difficulty. In Area v, there was no significant difference in skill level. However, a Kruskal-Wallis test detected significance in Task difficulty (*p* < .001). The time proportion of the Easy Task was significantly shorter than the time spent on the Moderate Task or the Hard Task (Wilcoxon Rank-Sum test, *p* < .001 and *p* = .014, respectively). In Area vi, there was no significant difference in skill level or Task difficulty.

P16L274: State what the finding was? Who had the higher proportion of saccades?

**Response:** Sorry for the lack of description. We have added the finding briefly as follows:

**Change:** [P17L294-298] For saccade percentage, a significant main effect of skill level was found (F = 8.07, p = .007, partial η^2^ = .15). There was no significant main effect of Task difficulty, nor interaction between skill level and Task difficulty. These results indicate that the saccade percentage of Expert players was significantly higher than that of Low Skill players, irrespective of Task difficulty.

**DISCUSSION:**

P1L299: This was not your purpose. Your purpose was to examine and compare the gaze control strategy between novices and expert SC players. Please rephrase.

**Response:** Thank you for your suggestion. This sentence has been rephrased.

**Change:** [P18L320-321] The purpose of this study was to examine the gaze control strategy of the esports experts (Expert) during a game which requires multitasking abilities, by comparing Expert with lower skilled players (Low Skill).

P17L300-301: The authors state ‘The experts showed significantly higher performance scores than the novices in all three Tasks, as expected. Therefore, it seems that the three Tasks modeled successfully discriminated between the abilities of the two subject groups’. This was not the case. Skill level differences could not be found in the easy task. Why might experts and novices differ only for moderate and hard tasks. Discussion should be provided around this.

**Response:** We apologize for the incorrect statement. We have removed “Easy Task” in this sentence and added a possible reason why there was no significant difference in performance in the Easy Task between expert and low skill players.

**Change:** [P19L323-324] Since the number of jobs that participants had to perform simultaneously in the Easy Task was small, the Easy Task was likely so easy that it was unable to reveal group differences, probably due to a ceiling effect.

P18L312-313: I would like the authors to speak to their interpretation that ‘the ratio of saccade was larger in the experts than in the novices, suggesting that e-sports experts frequently use saccade for quickly obtaining the information they need.’ This seems to contradict previous literature that suggests information is not gathered during saccades but during fixations. They seem to note this when discussing their fixation ratio findings. I would like the authors to contrast this with expertise literature on quiet eye etc., which would suggest that longer final fixations durations are a marker of expertise.

**Response:** We appreciate your helpful comment and apologize for the unclear expression that induces a misunderstanding. As you noted, information cannot be gathered during saccades. Thus, we believe that esports experts frequently and effectively use saccade to quickly move the gaze, and judge information at the location where the saccade arrives. We have added the descriptions to contrast our findings in esports with some papers on gaze movements during general sports.

**Change:** [P19L337-338] Second, the ratio of saccade was larger in the Expert than in the Low Skill players, suggesting that esports experts frequently use saccade to quickly process multiple stimuli.

**Change:** [P21L360-372] It is well known that skilled athletes in general sports also show different gaze behavior from novice or low skill players. For example, in one-on-one defensive situations in soccer, novice players mostly watch the ball, while experienced players watch not only the ball but also the knee and hip of the opponent player [5], probably because experienced players pay attention more broadly to the overall movement of their opponent. Similarly, in RTS players must pay attention to multiple stimuli. The current study may expand the knowledge of gaze control strategy during sports, i.e., the strategy of distributing the gaze to wide areas in the visual field might be common when players in either general sports or esports need to pay attention to multiple information streams simultaneously. Meanwhile, it has been proposed that athletes in general sports use different gaze control strategies depending on the situation [26]. For example, elite basketball players fix their gaze on the hoop significantly longer than novices do in a free throw situation [21]. In this case, throwing the ball into the hoop is the only requirement, so it is advantageous for players to fix their gaze on the hoop. From the above, we can estimate that esports players would probably also utilize different gaze strategies depending on the game genre. However, this remains to be elucidated in future studies.

P18L326: Starcraft is a RTS, not an AVG. Please revise here and throughout.

**Response:** Thank you for this comment. Now we have changed AVG to RTS in the Discussion section.

P19L331-333: This argument that experts direct focus to the foot in soccer does not support the authors claim that displaying more saccades across a larger horizontal field of view is a marker of expertise. In fact, it suggests the opposite, that experts in traditional sports maintain focus on a small area whereas experts in esports do not. I hope they might be able to clarify and add to their argument here.

**Response:** We really appreciate your suggestion. We have modified and added explanations regarding the difference and commonality between esports and general sports as follows:

**Change:** [P21L360-370] It is well known that skilled athletes in general sports also show different gaze behavior from novice or low skill players. For example, in one-on-one defensive situations in soccer, novice players mostly watch the ball, while experienced players watch not only the ball but also the knee and hip of the opponent player [5], probably because experienced players pay attention more broadly to the overall movement of their opponent. Similarly, in RTS players must pay attention to multiple stimuli. The current study may expand the knowledge of gaze control strategy during sports, i.e., the strategy of distributing the gaze to wide areas in the visual field might be common when players in either general sports or esports need to pay attention to multiple information streams simultaneously. Meanwhile, it has been proposed that athletes in general sports use different gaze control strategies depending on the situation [26]. For example, elite basketball players fix their gaze on the hoop significantly longer than novices do in a free throw situation [21]. In this case, throwing the ball into the hoop is the only requirement, so it is advantageous for players to fix their gaze on the hoop.

**FIGURES:**

Figure 4: incorrect reporting of statistical findings. Firstly, graphs should be shown as two plots (one comparing Novice to expert with data pooled for difficulty in the two bars – then a significance star between the two bars to show the main effect of skill level. Then the second plot, 3 bars with expertise pooled in each of the easy, moderate and hard bars, with star over easy to show it was different from the other two bars. The vertical graph can be separated into two plots in the same way, such that you have figures 4a, 4b, 4c, and 4d with a and b plots showing horizontal data and c and d plots showing vertical data.

**Response:** Thank you for your advice on figures. Accordingly, Figure 4 has been separated into two different plots (Skill level and Task difficulty) and the labeling of each plot has been changed to a, b, c, and d.

Figures 5, 6 and 7 can all be adapted in the same way. If the authors feel strongly about presenting the individual skill level and expertise bars, I would recommend the presentation of the data in this way be done in the form of supplementary material figures. The figures as they are, are not effective in their display of the main findings and support for or against the purpose and hypotheses laid out in this manuscript.

**Response:** Thank you for your consideration. We think that following your recommendations is a more effective way to explain our findings. Figure 5, 6, and 7 have been edited like Figure 4 was (separated into two plots and a changed labeling method).

Figure 5

Figure 6

Figure 7

**Reviewer#2:**

**General comments:** Thank you for your manuscript that investigates the difference in aspects of gaze control between experts esports players and novices while playing the game StarCraft. However, I will address major concerns that could encourage authors to improve the manuscript.

**Response:** We sincerely appreciate your careful review and helpful comments. We hope the manuscript will be improved with this revision.

First of all, the introduction section needs some rework, starting from implementing or acknowledging the current standardise definitions and conceptualisation of esports. This, as authors are using a wrong spelling of the word esports and it is important to clarify that esports is not a subcategory of action video games (AVG) (see review, (Pedraza-Ramirez et al., 2020).

**Response:** Thank you for your suggestion. Definitions and conceptualization of esports have been edited. Additionally, we are sorry for spelling esports incorrectly. Now, “e-sports” has been changed to “esports” and “Esports”. We understand that esports is not a subcategory of action video games. We have shown the difference between them as follows:

**Change:** [P2L35-36] Irrespective of game genre, when a game is played competitively and professionally, it can be considered esports [2].

Even though authors are acknowledging the current literature of video games and cognitive functions, it is recommended to consider including not only the single studies in the video gaming literature but as well the recent meta-analysis that have found mixed evidence on the effects of video game practice on cognition (e.g., Bediou et al., 2018; Sala et al., 2017) and the current state of the art and relevant studies in esports (e.g., Li et al., 2020; Pedraza-Ramirez et al., 2020; Thompson et al., 2019; Toth et al., 2019).

**Response:** Thank you for your suggestion about references. We carefully checked your reference list and decided to add the references about meta-analysis of esports (Li et al. 2020) and related research about esports (Bediou et al. 2018).

**Change:** [P3L40-44] For example, Kowal et al. [16] found that the task-switching ability of action video game (AVG) players is higher than that of non-AVG players. Furthermore, top video game players have better cognitive flexibility than novice players [15] in their ability to adapt to new and unexpected conditions [7]. In addition, a recent meta-analysis also found that video game practice has positive effects on cognitive function [14] (the mental processing required to obtain information and knowledge [6]).

In general, the manuscript needs to improve at the theoretical level. This, because it was not mentioned any theoretical background that can help to support the aim of the study and develop clear assumptions. Authors identified different areas of investigation in relation to the aim of the study such as cognitive functions, multitasking or motor-cognitive processes, however, it is not clear the theoretical foundation. However, due to the lack of specificity the manuscript is not doing a good job showing how this research will contribute to the extension of knowledge. Even though it was shown in the manuscript a great number of studies supporting the importance to study visual functions and saccade control ability in video game players, authors need to consider, first, a theoretical account that will help to support the hypothesis. Second, acknowledging the considerations towards the issues of lack of rigorous methodological designs in video game research and esports (e.g., not grouping video game genres) (see Dale & Green, 2017). Third, differentiating and bring specificity whether the study is on esports or video games. Consequently, the current state of the hypothesis is too vague and do not have a supporting evidence, thus, affecting the trustworthiness of the study.
We are thankful for your comment. We have tried to refocus the theoretical underpinnings of our manuscript and have added some comments on how this study contributes to an extension of our knowledge.

**Response to the first point (a theoretical account that will help to support the hypothesis):**

Thank you for your suggestion. We have modified the sentences explaining the theoretical background for setting the hypothesis as follows:

**Change:** [P3L45-P4L61] The real-time strategy (RTS) game is another kind of video game. In RTS games players are required to multi-tasking using complex strategies. AVG and RTS have similar positive effects on cognitive processes (both AVG and RTS improve reaction time and problem-solving ability) [10]. Meanwhile, Dobrowolski et al. [4] found that RTS players have superior cognitive function compared to that of AVG players. Indeed, RTS game training improves task switching ability [18]. In order to switch between tasks, it is necessary to accurately perceive stimuli presented on the monitor screen through proper gaze control.

Indeed, in RTS, multiple information streams appear simultaneously in different locations on the monitor screen, and players must absorb and select information of importance to them. Based on the information obtained, they must judge how to make an appropriately timed response to the stimuli. Thus, multitasking ability could be the key element underlying higher performance in RTS. RTS players must move their gaze over multiple areas on the monitor to win the game. Therefore, esports players would use more ballistic gaze movements, called saccade, to enable them to process multiple stimuli more quickly. However, the characteristics of gaze strategy in esports players are still unknown. Therefore, the purpose of the current study was to examine the gaze control of RTS expert players. We hypothesized that RTS experts in esports would show superior gaze control strategy (wide and fast saccadic gaze movement) which is evaluated by measuring gaze distribution and saccadic movements. Clarifying the gaze control strategy used by successful esports players would be beneficial for esports coaching, and also for expanding esports markets.

**Response to the second point (lack of rigorous methodological design):**

Thank you for advice about methodological designs in video game research and esports. Unfortunately, we did not collect the StarCraft players’ play history of another genre of video game. Thus, we cannot rule out the possibility that other esports play history have created the difference between the Expert and the Low Skill players. This information has been added in the limitation section as follows:

**Change:** [P22L393-395] In this study, we did not record the participants’ experience in playing other esports or their years of esports experience. Therefore, we cannot rule out the possibility that a history of playing other esports affected the difference in gaze movement between the Expert and the Low Skill players.

**Response to the third point (differentiating and bringing specificity to whether the study is on esports or video games.):**

We apologize for unclear expressions. The focus of the current study is how the ability and strategy in high level esports players differ from those of people who just enjoy video games.

**Response to all three comments:**

In response to your opinion that our hypothesis is too vague, we have modified the description of how the hypothesis was created as follows:

**Change:** [P3L51-P4L61] Indeed, in RTS, multiple information streams appear simultaneously in different locations on the monitor screen, and players must absorb and select information of importance to them. Based on the information obtained, they must judge how to make an appropriately timed response to the stimuli. Thus, multitasking ability could be the key element underlying higher performance in RTS. RTS players must move their gaze over multiple areas on the monitor to win the game. Therefore, esports players would use more ballistic gaze movements, called saccade, to enable them to process multiple stimuli more quickly. However, the characteristics of gaze strategy in esports players are still unknown. Therefore, the purpose of the current study was to examine the gaze control of RTS expert players. We hypothesized that RTS experts in esports would show superior gaze control strategy (wide and fast saccadic gaze movement) which is evaluated by measuring gaze distribution and saccadic movements. Clarifying the gaze control strategy used by successful esports players would be beneficial for esports coaching, and also for expanding esports markets.

Additionally, the method section needs rework, as the procedure is not clearly explained step by step for replicability and is missing important information. For example, we do not know whether was controlled that participants were engaging in other esports or video games or if it was asked for the years of experience playing StartCraft or other games. This, as experience and the type of game played may be crucial elements when investigating expertise differences in relation to cognitive abilities (see Li et al., 2020). Also, authors are not showing an a priori G* Power calculation to estimate the sample size required. Thus, clarifying and addressing the reliability of the methods used, clarity in the participants' background, and the procedure of the data collection brings rigour and reliability of the methodological design. Although the experimental task is very innovative and can add methodological value to research and its applicability in esports due to its ecological validity, I am wondering whether the test is supported by a previous investigation or if it was previously piloted, and if so, it would be required to address the findings so that there are not risks of construct validity. Lastly, I think that they behavioural data could be interesting form a motor learning and motor control perspective, however, I am wondering how this data aligns with the aim of the study. Therefore, integrating the results from this performance variable into the characteristics of gaze control could bring a better understanding of expertise in this manuscript.

**Response to the first point (replicability and missing information):**

Thank you for your comments. We agree that detailed information is lacking in the methods section and have added information about the participants as follows:

**Change:** [P22L393-395] In this study, we did not record the participants’ experience in playing other esports or their years of esports experience. Therefore, we cannot rule out the possibility that a history of playing other esports affected the difference in gaze movement between the Expert and the Low Skill players.

**Response to the second point (result about G*power analysis):**

We apologize for omitting information about the G*power analysis. We calculated the G*power using preliminary experimental data which compared gaze movement of esports expert and low skill players. Specific information about the G*power calculation has been added as follows:

**Change:** [P5L78-84] Before starting the experiment, we performed a power analysis to estimate the required sample size (RStudio version 1.3). The sample size was calculated using preliminary experimental data related to the distribution of gaze movements of esports experts (Expert) and players with lower skills (Low Skill) (effect size: 0.51; α level: 0.05; power (1-β error probability): 0.80). As a result, the calculated necessary number of participants was seven in each group. According to the result of this power analysis, sixteen participants (15 male, 1 female; 7 Expert, 9 Low Skill; mean age, 22.4 years; age range, 18-28 years) with experience playing StarCraft participated in the present study.

**Response to the third point (construct validity 1):**

Thank you for your concern about the construct validity of our study. The tasks used in the current study are original and are supported by our preliminary investigations and previous research about RTS (Dobrowolski et al., 2014 and Basak et al., 2014). All participants agreed that our tasks required a high level of multitasking ability. Information about preliminary investigations and our reason for using an original task have been added as follows:

**Change:** [P4L62-67] Murphy and Spencer [17] argue that there was no significant difference between esports players and non-esports players in visual attention skill; this conclusion was verified by using only a simple attention blink task in a laboratory, which was unrealistic because there was no multitasking involved. Therefore, in the current study for analyzing how esports players control their gaze while playing a game, we set them a task which was similar to what occurs in real RTS games; we required players to exhibit higher multitasking abilities, and to cope with increasing task difficulty.

**Change:** [P10L167-169] Each Task was original and conducted in response to preliminary investigations measuring gaze movement in esports experts. Participants in our preliminary investigations agreed that a high level of multitasking abilities was required for each Task.

**Response to the fourth consideration (construct validity 2):**

The reason why we used our original task is that previous research papers have only verified the cognitive function in RTS players by using simple laboratory tasks. To be specific, Murphy and Spencer (2009) argue that there was no significant difference between esports players and non esports players in visual attention skill. However, visual attention skill was only verified using a simple attention blink task. Thus, we thought it necessary to focus on the gaze movement of RTS players in actual video game circumstances with a higher difficulty level and closer resemblance to reality.

**Change:** [P3L54-P4L67] RTS players must move their gaze over multiple areas on the monitor to win the game. Therefore, esports players would use more ballistic gaze movements, called saccade, to enable them to process multiple stimuli more quickly. However, the characteristics of gaze strategy in esports players are still unknown. Therefore, the purpose of the current study was to examine the gaze control of RTS expert players. We hypothesized that RTS experts in esports would show superior gaze control strategy (wide and fast saccadic gaze movement) which is evaluated by measuring gaze distribution and saccadic movements. Clarifying the gaze control strategy used by successful esports players would be beneficial for esports coaching, and also for expanding esports markets.

Murphy and Spencer [17] argue that there was no significant difference between esports players and non-esports players in visual attention skill; this conclusion was verified by using only a simple attention blink task in a laboratory, which was unrealistic because there was no multitasking involved. Therefore, in the current study for analyzing how esports players control their gaze while playing a game, we set them a task which was similar to what occurs in real RTS games; we required players to exhibit higher multitasking abilities, and to cope with increasing task difficulty.

**Response to the fifth consideration (relationship between behavioral data and gaze movement):**

Thank you for the comment. As described in the manuscript, our results suggest that esports experts show superior ability both from behavioral data (game performance) and gaze control parameters (horizontal gaze distribution, saccade percentage, saccade velocity, and saccade number). As for the relationship between them, some gaze control parameters showed significant correlation with behavioral data, but this significant correlation was not observed in every parameter, probably due to the small sample size. Please check the following graphs.

Finally, it is necessary to strengthen and highlight the added values and extension of knowledge at the introduction and support it in the discussion. The discussion section will benefit of integrating supporting evidence align with the findings. I think authors are doing a good job in lines 324 to 339 by discussing the results with previous evidence and expanding the knowledge.

The comments are mean to encourage authors to improve the quality and specificity of the direction of the manuscript.

**Response:** Thank you for your positive comment about our manuscript. We believe our revised manuscript is stronger than the original, and we highlight our findings as follows:

**Change:** [P21L360-372] It is well known that skilled athletes in general sports also show different gaze behavior from novice or low skill players. For example, in one-on-one defensive situations in soccer, novice players mostly watch the ball, while experienced players watch not only the ball but also the knee and hip of the opponent player [5], probably because experienced players pay attention more broadly to the overall movement of their opponent. Similarly, in RTS players must pay attention to multiple stimuli. The current study may expand the knowledge of gaze control strategy during sports, i.e., the strategy of distributing the gaze to wide areas in the visual field might be common when players in either general sports or esports need to pay attention to multiple information streams simultaneously. Meanwhile, it has been proposed that athletes in general sports use different gaze control strategies depending on the situation [26]. For example, elite basketball players fix their gaze on the hoop significantly longer than novices do in a free throw situation [21]. In this case, throwing the ball into the hoop is the only requirement, so it is advantageous for players to fix their gaze on the hoop. From the above, we can estimate that esports players would probably also utilize different gaze strategies depending on the game genre. However, this remains to be elucidated in future studies.
