# Supplementary material for: Difference in gaze control ability between low and high skill players of a real-time strategy game in esports
Source: PLoS One. 2022 Mar 18;17(3):e0265526. doi: 10.1371/journal.pone.0265526 (PMC8933040; doi:10.1371/journal.pone.0265526)
Supplement: Attachment — Submitted filename: response letter_R2.docx [file pone.0265526.s008.docx]

**Response to Reviewers**

Dear Reviewers

Thank you very much for your feedback, comments, and suggestions. Our responses to your comments are as follows:

**Reviewer #1:**

**General comment:** Many thanks to the authors for addressing my initial comments.

The paper has improved significantly, but I do feel requires a number of revisions and amendments still before meriting publication. My comments are listed below.

**Response:** Thank you for your general comment. We hope we have improved our manuscript through this revision.

**Title:** As your paper address the difference in gaze control ability between low and high skill RTS players, the title should be revised to reflect this more accurately. I suggest ‘Gaze control ability between low and high skill players of a real-time strategy game.

**Response:** Thank you for your suggestion. We decided to follow your suggestion about the title. Thus, we changed the title ‘Gaze control ability in esports expert’ to ‘Difference in gaze control ability between low and high skill players of a real-time strategy game in esports’.

**Change:** [P1L3-4] **Difference in gaze control ability between low and high skill players of a real-time strategy game in esports.**

**Abstract:** You often interplay between multitasking and task-switching ability throughout the manuscript. I would argue that your data are attempting to support the latter as humans generally cannot multitask and what we might think as multitasking has been shown to just be evidence of superior task switching ability. I would suggest the authors replace multitasking with task-switching throughout the manuscript.

**Response:** We appreciate your comments. We changed the word ‘multitasking ability’ to ‘task-switching ability’ in the abstract and throughout the manuscript.

**Change:** [P2L28-29] This could be one of the factors enabling esports experts to perform better than players with lower skills when playing games that require task-switching ability.

**Introduction:**

Line 36: Esports are the competitive and often professional play. They don’t have to be professional to be esports. Revise the sentence to amend the wording please.

**Response:** Thank you for your comment. We revised the wording in this sentence.

**Change:** [P2L35-36] Irrespective of their genre, esports are very competitive and often professionally played.

Line 45-46: Change multitasking to multitask.

**Response:** Thank you. The word ‘multitasking’ has been changed to ‘task-switching’ and is used consistently throughout the manuscript.

**Change:** [P3L46] In an RTS game, players are required to task-switch using complex strategies,

Lines 46-47: Change ‘(both AVG and RTS improve reaction time and problem-solving ability)’ to ‘(playing either AVG and RTS games improves reaction time and problem-solving ability)’

**Response:** Thank you for your suggestion. The sentence in this section has been entirely changed.

**Change:** [P3L49-51] It is probable that considering these characteristics of an RTS game, positive effects of playing RTS games on cognitive processes (reaction time and problem-solving ability) occur [10].

Line 55: Change saccade to saccades

**Response:** We appreciate the suggestion. The word ‘saccade’ has been changed to ‘saccades’.

**Change:** [P4L62] saccades,

Line 58: By ‘wide’ do you mean dispersed? This might be a better word. Also, I would assume it is the fixations and not saccades over a larger area that is a marker of expertise? So faster saccades and a larger area over which fixations are dispersed? If so, please amend.

**Response:** Thank you for your consideration. We assumed that a larger fixation area is a marker of expertise. Thus, the wording about ‘wide’ has been changed to ‘dispersed’.

**Change:** [P4L63-64] (dispersed and fast saccadic gaze movement).

Lines 60-61: why would clarifying the gaze control strategy used by successful esports players be beneficial for expanding esports markets? Please clarify in the manuscript.

**Response:** Thank you for your opinion. We deleted the sentence which mentioned expanding the esports market, because it has essentially no relationship to our research. Also, the sentence about esports coaching has been moved to the discussion section.

**Change:** [P22L392-393] Additionally, knowledge about the gaze control strategy used by successful esports players would be helpful for esports coaching and for devising new training methods.

Lines 62-67: This paragraph creates confusion as everything you have leading up to the study’s purpose centers around multitasking/task switching ability. I understand that attention is relevant here especially when looking t fixations, but this paragraph needs to come earlier and be part of the setup to the purpose, not after it. Please amend.

**Response:** Thank you for your suggestion. The content of lines 62-67 in the previous version has been removed because another reviewer claimed that this previous research lessened the strength and validity of the current study. Additionally, a new hypothesis and background of our research has been added (P3L46-P4L65).

Line 68: Starcraft needs to be introduced earlier. Again, everything should be setup before the purpose so when it is introduced at the very end of the introduction section, it seems obvious to the reader that the purpose is what you say it is, then follow the purpose with your hypotheses to conclude your introduction.

**Response:** We entirely agree with your opinion. We introduce StarCraft earlier and it is now explained before the purpose section **(**P3L46-P4L65**)**.

**Method:**

Lines 78-84: Please provide your power analysis in your reply here. Why use Rstudio and not Gpower, which is the typical standard. Given you are using prelim experimental data, I assume you have means and SD’s you are inputting for your calculations? Please provide these. Finally, your power analysis should be outputting equal sample sizes between your groups which may or may not match what you have collected exactly. As such, I don’t believe the power analysis was conducted properly and I think a supplementary file or some file here demonstrating the raw inputs and outputs from the power analysis is needed to verify the study is appropriately powered to detect the differences found.

**Response:** Thank you for your comment. There was no specific reason why we used RStudio. Thus, we calculated the required sample size using G*Power software. G*Power was calculated by using the Hard Task performance level of experts (Expert, n = 4) and players with lower skills (Low Skill, n = 4) of our preliminary experiment. In the main experiment we used the different sample size for the two groups, because it was easy to recruit Low Skill subjects. Raw input and output of the power analysis is as follows:

Raw data of each participant: Expert (224, 212, 153, 225), Low Skill (146, 176, 113,141)

Power calculation for unpaired t-test between Expert (n = 4) vs. Low Skill (n = 4)

t test: Means: Difference between two independent means (two groups)

Analysis: Compute required sample size

Input: Tail(s) = Two

Effect size d = 1.91

α err prob = 0.05

Power (1-β err prob)) = 0.9

Allocation ration N2/N1 = 1

Output: Noncentrality parameter δ = 3.57

Critical t = 2.17

Df = 12

Sample size group 1 = 7

Sample size group 2 = 7

Total sample size = 14

Actual power = 0.905

Specific G*Power protocols have been provided in Supplement file S6.

**Change:** [P4L72-P5L77] We performed a power analysis to estimate the required sample size (G*Power version 3.1). The G*Power was calculated using the Hard Task performance level (described in the Sample games section) of experts (Expert, n = 4) and the performance of players with lower skills (Low Skill, n = 4) in a preliminary experiment (Cohen’s d: 1.91; α level: 0.05; power (1-β error probability): 0.9). Effect size was calculated according to Cohen [17]. The results showed that seven participants were required for each group. The details of the G*Power protocol are presented in the S6 Supplement file.

Line 97: Same comment as above. The jobs are not performed simultaneously but require very rapid and accurate task switching ability. Please amend.

**Response:** Thank you for this comment. The word ‘multitasking’ has been changed to ‘task-switching’.

**Change:** [P6L103-104] In this process a high level of task-switching ability is required to perform all these jobs simultaneously.

Line 127-128: Watch grammar and make sure tense is kept consistent. The sentence “The difficulty of each Task depends on how many jobs are required and how many Zones are in play at the same time” should read “The difficulty of each Task depended on how many jobs were required and how many Zones were in play at the same time”. This is important and needs to be proof-read and corrected throughout the manuscript to improve readability.

**Response:** Sorry for grammatical error of our manuscript. We revised the content of Line127-128 and the whole manuscript.

**Change:** [P8L133-135] The difficulty of each task depended on how many jobs were required and how many Zones were in play at the same time. When more jobs were required and more Zones were used at the same time, the difficulty of the task increased.

Line 177-178: The equation you present equally weights the sum of buildings constructed and units produced, and the enemy units destroyed. To more strongly emphasize the units destroyed, sum the two of these together but multiply each by a constant that is larger for enemy units destroyed.

i.e., (units produced + buildings constructed)*A + (enemies destroyed)*B, where B > A

**Response:** Thank you for suggestion. We followed your suggestion and reworked the performance level.

**Change:** [P11L178-179] *(number of constructed buildings + number of produced units) ×2+ (number of destroyed enemy units) ×3*

The reason for using different constants is to strongly emphasize the number of destroyed enemy units.

**Change:** [P15L255-257] (Moderate Task: mean 170.28 ± SD 19.20 vs. mean 137.22 ± SD 25.97, t = 3.31, df = 15.89, *p* = .004, and Hard Task: mean 208.14 ± SD 26.01 vs. mean 155.55 ± SD 37.26, t = 3.75, df = 15.71, *p* = .001)

Lines 236-237: Change ‘calculated’ to analyzed.

**Response:** Thank you. The word ‘calculated’ has been changed to ‘analyzed’

**Change:** [P14L237-238] For this reason, these data were analyzed by a non-parametric method.

Line 237-239: change “When the Kruskal-Wallis test detected significance, the Wilcoxon Rank-Sum test was performed to find the difference between each group of Task difficulties (Bonferroni’s correction (p < .05 divided by 3 tests: significance threshold at .017)).”

To

“When the Kruskal-Wallis test detected significance, the Wilcoxon Rank-Sum test was performed to determine which Task difficulties were significantly different from one another (Bonferroni’s correction (p < .05 divided by 3 tests: significance threshold at .017)).”

**Response:** Thank you for this correction. The sentence ‘find the difference between each group of Task difficulties’ has been changed to ‘determine which Task difficulties were significantly different from one another’.

**Change:** [P14L238-L240] When the Kruskal-Wallis test detected significance, the Wilcoxon Rank-Sum test was performed to determine which Task difficulties were significantly different from one another (Bonferroni’s correction (*p* < .05 divided by 3 tests: significance threshold at .017)).

**Result:**

Lines 299-302: you report finding main effects for skill and task difficulty, with no interaction. However, your post hoc reports an interaction in that low skill participants had higher fixation percentage in the moderate compared to easy task. Please revise your reporting here as the main effect suggests you should only be reporting expertise differences whereby all task difficulties are pooled, OR, reporting differences between task difficulties, whereby all expertises within each task difficulty are pooled.

**Response:** We apologize for our statistical error. Our statistics reporting has been edited throughout the entire manuscript.

**Change:** [P15L268-P16L270] Simple main effect analysis found that SD of horizontal gaze distribution during the Easy Task was higher than that during the Moderate Task and Hard Task, irrespective of skill level (*p* < .001 for both).

**Change:** [P17L301-303] Simple main effect analysis indicated that the fixation percentage in the Moderate Task was significantly higher than in the Easy Task, irrespective of skill level (*p* = .02).

Line 333-334: Please change

‘Thus, when playing such games, it is necessary to simultaneously pay attention to multiple points and areas on the screen.’

to

“Thus, when playing such games, it is necessary to rapidly switch attention to multiple points and areas on the screen and maintain this information in working memory.’

**Response:** Thank you for this correction. The text has been corrected as pointed out.

**Change:** [P19L334-336] Thus, when playing such games, it is necessary to rapidly switch attention between multiple points and areas on the screen and maintain this information in working memory.

Line 338: Saccades are never used to process information. Information is gathered during fixations. Please amend the wording of this sentence to reflect this.

**Response:** Thank you for this correction. We understand saccades are never used to process information. Thus, we argued that saccades are used to pay attention to multiple points and areas on the screen.

**Change:** [P19L338-341] Second, the ratio of saccade was larger and fixation time was shorter in the Expert than in the Low Skill players, suggesting that esports experts frequently use saccade to quickly change their gaze position and accumulate more information through their shorter fixation time.

Line 346: The way this is written is counterintuitive. If Experts exhibit more saccades, they would also have more fixations. In your case, this is also true, but low skill individuals have a higher fixation time. So to say experts have more saccades but low skill players have a higher percentage of fixation could imply that low skill players have more fixations. Please amend wording to clarify that it is in fact that low skill individuals have longer fixation durations.

**Response:** Thank you for this advice. We amend the wording to clarify that the Low Skill players have longer fixation durations.

**Change:** [P20L348-350] Finally, the higher percentage of fixation in the Low Skill than in the Expert players (Figure 7c and 7d) indicates that the Low Skill players needed longer fixation duration to get information and therefore needed to keep their gaze on one location longer.

Line 352: Change multitasking to task switching.

**Response:** Thank you. Multitasking has been changed to task-switching.

**Change:** [P20L354-355] These eye movements are advantageous to processing multiple stimuli and successfully playing games when task-switching ability is required.

**Reviewer #2:**

**General comment:** Thank you for resubmitting your manuscript and consideration of my general comments. The authors have addressed many of the suggestions, however, I will address some concerns that I still think need further attention.

**Response:** Thank you for your careful review and your advice. Our manuscript has been reworked according to your specific comments.

Overall, the manuscript presents improvements in the different sections where concerns were raised. However, the introduction still needs to be revisited as it reads too general and does not present a clear theoretical framework. A theory driven study will help the authors to propose hypotheses based on evidence and give clear direction to the further of knowledge.

**Response:** Thank you for your consideration. The Introduction section has been reworked according to your suggestions. Please check the revised Introduction parts, especially from the second to the fourth paragraph (P3L45-P4L65).

Additionally, there are a few inconsistencies while describing real time strategy games and action video games, esports and video games especially when referring to previous work (see specific comments).

**Response:** Thank you for comments on the usage inconsistences of RTS, video game, and esports. We checked through the manuscript and corrected these terms.

Furthermore, while the aim of the study is to examine gaze control of RTS expert players, first, it would be important to emphasise that the aim is directed towards StarCraft players and not the whole RTS genre;

**Response:** Thank you for this suggestion. We emphasized that this study analyzed StarCraft players to shed light on the characteristic of gaze movement in RTS players.

**Change:** [P3L55-57] In light of the above background, the current study aimed to clarify how RTS players control their gaze while playing a game. In order to shed light on their gaze control strategy, we adopted a popular RTS game, StarCraft, as the model task.

Second, the role of cognitive abilities needs to be clarified, more precisely those with greater importance for the aim of the study require further detail and argumentation. Even though the authors are briefly addressing different studies showing the benefits of esports on specific cognitive abilities, it is hard to follow the red threat on why multitasking is an important ability for esports performance and what is the connection with gaze behaviour. Consequently, the theoretical link and underlying mechanisms at the specific cognitive level are still missing.

**Response:** Thank you for this suggestion. We reworked the whole introduction section to explain the reasons for focusing on the gaze control ability and multitasking ability (we have changed “multitasking ability” to “task-switching ability” according to another reviewer’s comments).

Furthermore, there are still issues with justifying the sample size and the validity and reliability of the task. I have noted a few specific comments on these issues below.

**Response:** Thank you for your consideration. The sample size has been recalculated with G*Power software. We provide a detailed description regarding the protocol of this power analysis in the revised manuscript. We believe that this information can increase the validity and reliability of our research.

**Change:** [P4L72-P5L77] We performed a power analysis to estimate the required sample size (G*Power version 3.1). The G*Power was calculated using the Hard Task performance level (described in the Sample games section) of experts (Expert, n = 4) and the performance of players with lower skills (Low Skill, n = 4) in a preliminary experiment (Cohen’s d: 1.91; α level: 0.05; power (1-β error probability): 0.9). Effect size was calculated according to Cohen [17]. The results showed that seven participants were required for each group. The details of the G*Power protocol are presented in the S6 Supplement file.

Lastly, it is important to review the writing in some sections and the use of capital letters where it shouldn’t be.

**Response:** Thank you for your suggestion. ‘Task difficulty’ has been changed to ‘task difficulty’ in whole manuscript. Additionally, ‘Task’ has been changed to ‘task’ where the word needed to be revised. Additionally, ‘Screen Marker Calibration’ and ‘Eye Movement Detector’ have been changed to ‘screen marker calibration’ and ‘eye movement detector’

P3L45: Real time-strategy is not a video game genre and not a video game itself.

**Response:** Thank you for this consideration. We removed the description that Real-time strategy (RTS) is a video game genre.

P3 L45-47: Please rework this paragraph

**Response:** Thank you for your comment. This paragraph has been reworked.

**Change:** [P3L45-48] The real-time strategy (RTS) game is a popular esports genre. In an RTS game, players are required to task-switch using complex strategies, because multiple information streams appear simultaneously in different locations on the monitor screen, and players must absorb and select information of importance to them.

P3 L51-56: If the main arguments and justifications of the study are going in the direction of the role and importance of multitasking in RTS games, it is necessary to first, extend on the literature of multitasking and second, support these arguments with evidence. So far this section still not strongly supported by empirical evidence and lacks theoretical accounts that can help to justify this link with video games and esports.

**Response:** Thank you for this comment. Multitasking has been replaced with task-switching. Additionally, we failed to find evidence supporting our arguments (superior task-switching ability could be the key element underlying higher performance in RTS games). Thus, this paragraph has been entirely reworked and the argument about higher performance in RTS games has been deleted.

**Change:** [P3L45-56] The real-time strategy (RTS) game is a popular esports genre. In an RTS game, players are required to task-switch using complex strategies, because multiple information streams appear simultaneously in different locations on the monitor screen, and players must absorb and select information of importance to them. Based on the information obtained, they must judge how to make an appropriately timed response to the stimuli. It is probable that considering these characteristics of an RTS game, positive effects of playing RTS games on cognitive processes (reaction time and problem-solving ability) occur [10]. Dobrowolski et al. [4] found that RTS players have superior cognitive function compared to that of AVG players. Training with RTS games in elderly people improved task-switching [18]. This could be accompanied by or be based on improving gaze control ability which RTS requires; that is, for smooth task-switching, it is necessary to quickly process multiple sources of visual stimulation.

In light of the above background, the current study aimed to clarify how RTS players control their gaze while playing a game.

P 3 L58-61: The hypothesis does not seem to have a theoretical link still justifying why authors are expecting those findings. However, this hypothesis reads very simplistic, as it is well known that experts will outperform novices in specific tasks. Thus, the hypothesis in its current state does not add knowledge and still too vague.

**Response:** Thank you for your comment. We reworked the hypothesis to interrelate the theory and previous findings. To be specific, we added some reasons why task-switching ability and gaze control strategy were responsible for the high performance in the RTS game. We believe that it helps to make our hypothesis convincing.

**Change:** [P3L55-P4L65] In light of the above background, the current study aimed to clarify how RTS players control their gaze while playing a game. In order to shed light on their gaze control strategy, we adopted a popular RTS game, StarCraft, as the model task. Moreover, since task difficulty (i.e., number of tasks that players have to switch between) would be a critical factor for gaze control, it is necessary to clarify whether or not gaze control, while playing an RTS game, depends on task difficulty. Thus, we set the test games with the three different levels of difficulty. Considering the characteristics of RTS games (StarCraft in this case) requiring players to quickly move their gaze over multiple areas on the monitor, and the fact that long-term training in RTS games improves task-switching ability [18], elite StarCraft players would use more ballistic gaze movements, called saccades, to quickly process multiple stimuli. Thus, we hypothesized that RTS experts would show superior gaze control (dispersed and fast saccadic gaze movement) while playing games compared to the novices. We tested this hypothesis by measuring gaze distribution and saccadic movements.

P3 L60-61: The practical implications as mentioned here for esports coaching and expanding esports markets should be either supported in the introduction with theoretical justifications or addressed at the end of the manuscript supported by the findings.

**Response:** Thank you for your consideration. According to your comment, we have deleted the descriptions of esports markets. Further, the description of practical applications has been moved to the end of the introduction section with theoretical justifications.

**Change:** [P22L392-393] Additionally, knowledge about the gaze control strategy used by successful esports players would be helpful for esports coaching and for devising new training methods.

P3 L66-67: As the test seems to fulfil some elements of ecological validity, I would expect justification why multitasking and the increase of task difficulty can help to understand closer what the StarCraft players experience while playing the game. Consequently, support from ecological validity and representative designs studies could help to support the decisions on the difficulty of the task.

**Response:** Thank you for your suggestion. We explained the reason why task-switching was linked to task difficulty. However, the level of difficulty required to clarify the difference between an expert player and a lower skill player is unknown. Thus, we believe that it is important to explain the different requirement of each task.

**Change:** [P3L57-P4L59] Moreover, since task difficulty (i.e., number of tasks that players have to switch between) would be a critical factor for gaze control, it is necessary to clarify whether or not gaze control, while playing an RTS game, depends on task difficulty.

P3 L62-64: The investigation used as support for the current study Murphy and Spencer (2009) was a replication study from Green and Bavelier (2003) in which the sample were not esports players as you mention and the games, they are focusing on are not RTS but action games in which combines different games. Thus, this argumentation loses strength and validity when the full story is not explained. The aim of these previous studies should mention and fully considered when comparing the results, aiming to extends your research based on those findings.

**Response:** Thank you for your comment. We reworked this paragraph by using an RTS game characteristic. When playing an RTS game, players are required to task-switch using complex strategies, because multiple information streams appear simultaneously in different locations on the monitor screen. Long-term training of playing RTS can improve cognitive processing and task-switching ability. However, the gaze control strategy in RTS game players during an actual RTS game has never been investigated. This information (the characteristics of RTS games and the lack of knowledge of the gaze control strategy during an actual RTS game) will help to support our hypothesis.

**Change:** [P3L45-56] The real-time strategy (RTS) game is a popular esports genre. In an RTS game, players are required to task-switch using complex strategies, because multiple information streams appear simultaneously in different locations on the monitor screen, and players must absorb and select information of importance to them. Based on the information obtained, they must judge how to make an appropriately timed response to the stimuli. It is probable that considering these characteristics of an RTS game, positive effects of playing RTS games on cognitive processes (reaction time and problem-solving ability) occur [10]. Dobrowolski et al. [4] found that RTS players have superior cognitive function compared to that of AVG players. Training with RTS games in elderly people improved task-switching [18]. This could be accompanied by or be based on improving the gaze control ability which RTS requires; that is, for smooth task-switching, it is necessary to quickly process multiple sources of visual stimulation.

In light of the above background, the current study aimed to clarify how RTS players control their gaze while playing a game.

P3 L64-67: I am not sure if this is adding to the hypothesis. It seems that the authors are interested in investigating multitasking and coping mechanisms. The multitasking bit was mentioned earlier but the introduction lacks literature in this field and its importance for the study.

P5 L75: Please be consistent with the language or make sure that connection between gaze control and multitasking is clear. So far, it is confusing to understand which are/is the real aim of the study, as there are different concepts drop during the introduction and methodology. Thus, the theoretical account is not clear, and hypothesis are not supported theoretically or empirically.

**Response to the above two comments:** Thank you for this consideration. Multitasking has been changed to task-switching ability in this revision. In this paper, we directly focused on the gaze control strategy in RTS experts, rather than the task-switching. Superior gaze control in RTS experts was expected, because the task-switching ability, which is required for high RTS performance, would be supported by gaze control ability. In the revised manuscript, we cited the research which focused on task-switching ability to support our idea (Basak et al. 2008). They argue that playing an RTS game improves the task-switching ability of older adults. Thus, there is a possibility that task-switching ability is an important factor for RTS game players. However, whether gaze control ability supports task-switching ability has never been investigated. This is the reason why we focused on gaze behavior in the current study.

**Change:** [P4L60-65] Considering the characteristics of RTS games (StarCraft in this case) requiring players to quickly move their gaze over multiple areas on the monitor, and the fact that long-term training in RTS games improves task-switching ability [18], elite StarCraft players would use more ballistic gaze movements, called saccades, to quickly process multiple stimuli. Thus, we hypothesized that RTS experts would show superior gaze control (dispersed and fast saccadic gaze movement) while playing games compared to the novices. We tested this hypothesis by measuring gaze distribution and saccadic movements.

P5 L78-81: Which preliminary experimental data are you referring to? This needs to be further justified.

**Response:** We apologize for the missing information. In this paragraph, G*Power has been recalculated by using 4 Expert and 4 Low Skill participants’ performance level data from our preliminary investigation.

**Change:** [P4L72-P5L77] We performed a power analysis to estimate the required sample size (G*Power version 3.1). The G*Power was calculated using the Hard Task performance level (described in the Sample games section) of experts (Expert, n = 4) and the performance of players with lower skills (Low Skill, n = 4) in a preliminary experiment (Cohen’s d: 1.91; α level: 0.05; power (1-β error probability): 0.9). Effect size was calculated according to Cohen [17]. The results showed that seven participants were required for each group. The details of the G*Power protocol are presented in the S6 Supplement file.

P5 L84-91: The are still information missing. For example, the recruitment strategy and the inclusion/exclusion criteria are still missing.

**Response:** Thank you for your comment. Information about recruitment strategy and the inclusion/exclusion criteria have been added.

**Change:** [P4L79-80] Participants were recruited through the Waseda University school bulletin board. Subjects had no record of visual disorders, and were excluded if they had less than six months experience playing StarCraft.

P9 L152-153: I think that the authors should allocate a section where the procedure of the experiment is explained in detailed. Additionally, a figure could help to show the overview of the experimental procedure and measures. This, as I see a critical issue in terms of the procedure of the study as there was not randomization of the difficulty of the tasks.

**Response:** Thank you for your consideration. We have added a procedure section and a figure showing the overview to our manuscript. We believe that this section will make it easy for readers to understand the experimental procedure as well as the existence of a limitation regarding non-randomization of the difficulty of the tasks.

**Change:** [P5L90-96] **Experimental procedure**

Before the experiment, we measured the distance at 40 inches between the subject’s head and the monitor and asked subjects to maintain the position during the task. Each task was performed for 3 minutes. When the participant failed to play the task for 3 minutes, the task was restarted. Additionally, any task which was not played for 3 minutes was excluded from the analysis. During each task, gaze movement was recorded by an eye tracker (Pupil Core, Pupil Labs). The tasks proceeded in the following order: Easy Task, Moderate Task, and Hard Task. When a task was performed for 3 minutes, the task was over.

**Change:** [P6L103] **Figure 1. Overall flow of the task.** Each step shows the overall flow of the task.

P10 L167-169: The preliminary investigations that you are referring to support the sample size and the reliability and validity of the task need to be further described and adequately referenced.

**Response:** Thank you for this consideration. In the preliminary investigation, four Expert and four Low Skill samples were used to estimate the required sample size. Also, the performance level of the Hard Task in the preliminary investigation was used to estimate the sample size. Specific data about the required sample size was as follows:

Raw data of each participant: Expert (224, 212, 153, 225), Low Skill (146, 176, 113,141)

Power calculation for unpaired t-test between Expert (n = 4) vs. Low Skill (n = 4)

t test: Means: Difference between two independent means (two groups)

Analysis: Compute required sample size

Input: Tail(s) = Two

Effect size d = 1.91

α err prob = 0.05

Power (1-β err prob)) = 0.9

Allocation ration N2/N1 = 1

Output: Noncentrality parameter δ = 3.57

Critical t = 2.17

Df = 12

Sample size group 1 = 7

Sample size group 2 = 7

Total sample size = 14

Actual power = 0.905

P11 L189: How did you control for the use of different settings of keyboard and mouse? And how this decision could influence the results of the test.

**Response:** Thank you for the comment. Each participant used their own keyboard and mouse. However, all keyboard and mouse settings were the same for all subjects. Thus, we believe that this difference did not influence the results of experiment.
