# Supplementary material for: Difference in gaze control ability between low and high skill players of a real-time strategy game in esports
Source: PLoS One. 2022 Mar 18;17(3):e0265526. doi: 10.1371/journal.pone.0265526 (PMC8933040; doi:10.1371/journal.pone.0265526)
Supplement: Attachment — Submitted filename: resoponse letter_R3.docx [file pone.0265526.s009.docx]

**Response to Reviewers**

Dear Reviewers

Thank you very much for your feedback, comments, and suggestions. Our responses to your comments are as follows:

**Reviewer #1:**

The authors should be commended for their work in addressing the comments. The manuscript is now ready for publication and I have recommended the manuscript be accepted pending a few small comments.

**Response:** Thank you for your general comments. Your comments were very helpful to improve

our manuscript.

Firstly on line 72-73, GPower is a software and thus is not calculated. Please amend this sentence.

**Response:** Thank you for your comment. We have amended this sentence in response to your comment.

**Change:** [P5L74-77] The power analysis was conducted using the Hard Task performance level (described in the Sample games section) of experts (Expert, n = 4) and the performance of players with lower skills (Low Skill, n = 4) in a preliminary experiment (Cohen’s d: 1.91; α level: 0.05; power (1-β error probability): 0.9).

Secondly, and most importantly the grammar throughout the manuscript is still very poor and the authors are strongly encouraged to have a native English speaker review and revise the manuscript for technical grammar. This includes but is not limited to poor use of plural vs nonplural words in wrong instances and the use of past and present tenses in the same sentence. Having been immersed in the review of the manuscript, I found myself able to adapt to these shortcomings to review the scientific merit of the work, but I fear the general readership will have trouble with the readability of the manuscript in its current state.

**Response:** Thank you for your suggestion. According to your suggestion, the manuscript has been proofread by a native English speaker who is a professional scientific editor (as mentioned in the Acknowledgment section).

**Reviewer #2:**

Thank you for resubmitting your manuscript and consideration of my comments.
Overall, there are improvements to the manuscript and more consistency in language and justification of specific decisions. However,

P2 L34-36: Please use the current literature to justify concepts and definitions (see Pedraza-Ramirez et al., 2020).

**Response:** Thank you for your suggestion. We added the reference which you suggest.

**Change:** [P2L34-35] Today it is common to play a competitive game with other players online; this activity is called electronic sports (esports) [1].

P2 L37-40: Please include systematic reviews and/or meta-analysis to justify the interest in researching the cognitive elements of esports, rather than highlighting that there are cognitive benefits already seen by the reference used of 2018. This seems to be misleading as only until 2020 and 2021 did research in esports cognition start to be published. To see some examples that can serve as supporting references for video games cognition and esports cognition please see accordingly (Pedraza-Ramirez et al., 2020; Powers et al., 2013).

**Response:** Thank you for your suggestion. We have cited some meta-analysis research according to your suggestion.

**Change:** [P2L38-P3L41] In addition, meta-analysis revealed that playing a commercial video game improves the information-processing skills of players, which include task-switching ability and visual processing [1, 3]. Task-switching is defined as the ability to quickly alternate among multiple separate tasks [4].

P3 L55-57: The aim to use the esports StarCraft does not mean that you can generalise the investigation to other RTS games. This issue can be seen in studies investigating cognitive function in action video games or in more specific genres like MOBAs (i.e., League of Legends, Dota) where mixed evidence has been found (e.g., Boot et al., 2008, Kokkinakis et al., 2017), by generalising findings of the investigation in one esports to the whole game genre. Therefore, I recommend clarifying and keeping it consistent through the manuscript that the specific cognitive process is closely related to the demands of the esport under investigation without generalising the findings to other similar esport games. This, as supported and suggested by different authors in the video game and esports research (Power et al., 2013; Dale & Green, 2017; Pedraza-Ramirez et al., 2020).

**Response:** Thank you for your opinion. We modified the sentences to emphasize that our aim was directed towards StarCraft players without generalizing the findings to other esports games. Not only P3L56-57 but also in the entire Introduction and Discussion sections, we have changed the description that formerly generalized the current finding to all esports. Alternatively, we added a sentence regarding the possibility that our findings (StarCraft players have a specific gaze control strategy) may be generalizable to other similar esports games.

**Change:** [P3L56-57] In light of the above background, we used a popular RTS game, StarCraft, as a research model of RTS, and aimed to clarify how StarCraft experts control their gaze during their play to estimate their gaze control ability.

**Change:** [P22L393-396] Additionally, Dale and Green discovered that training using RTS games improves task-switching ability [12]. This suggests that our findings (StarCraft experts have a specific gaze control strategy) can be generalized to other esports games, and we believe that the specific gaze control strategy used by expert esports players is the source of their superior performance.

P4 L61-64: This claim shown as fact cannot be done by one individual study in an elderly population, additionally the study is from 2008 with very specific conditions. Therefore, the theoretical support for the hypothesis is still missing as currently there is no supporting evidence that justifies the expected expertise differences. Thus, I recommend you search in the recent reviews and empirical studies.

**Response:** Thank you for your suggestion. We have added the following references. Chiappe et al. (2013) argue that a first-person shooting (FPS) game improves task-switching ability, and according to Klaffehn et al. (2018), FPS players and RTS players have similar task-switching ability. To be specific, RTS players and FPS players have similar reaction times during task switching. Finally, not only a single case study, but also a meta-analysis suggests that RTS improves task-switching ability (Dale and Green, 2017). We believe these references provide supporting evidence that justifies the expected expertise differences.

**Change:** [P4L60-64] Additionally, it is already known that first-person shooting (FPS) games improve task-switching ability [14]; in addition, not only a single case study but also a meta-analysis study argues that RTS improves task-switching ability [11]. One study has found that FPS games and RTS games have a similar positive effect on task-switching ability [15]. Thus, elite StarCraft players should have superior task-switching ability.

**[References]**

11.	Dale G, Shawn Green C. The Changing Face of Video Games and Video Gamers: Future Directions in the Scientific Study of Video Game Play and Cognitive Performance. J Cogn Enhanc [Internet]. 2017;1(3):280–94. Available from: https://doi.org/10.1007/s41465-017-0015-6

14.	Chiappe D, Conger M, Liao J, Caldwell JL, Vu K-PL. Improving multi-tasking ability through action videogames. Appl Ergon [Internet]. 2013;44(2):278–84. Available from: https://www.sciencedirect.com/science/article/pii/S0003687012001202

15.	Klaffehn AL, Schwarz KA, Kunde W, Pfister R. Similar Task-Switching Performance of Real-Time Strategy and First-Person Shooter Players: Implications for Cognitive Training. J Cogn Enhanc [Internet]. 2018;2(3):240–58. Available from: https://doi.org/10.1007/s41465-018-0066-3
